# Special Issue “Mitochondrial Respiration in Physiology and Pathology”

**DOI:** 10.3390/ijms25052958

**Published:** 2024-03-03

**Authors:** Angela Messina, Andrea Magrì

**Affiliations:** Molecular Biology Laboratory, Department of Biological, Geological and Environmental Sciences, University of Catania, Via S. Sofia 97, 95123 Catania, Italy

Mitochondria are key organelles that regulate several functions essential for maintaining cellular homeostasis. In addition to their involvement in apoptosis regulatory pathways and calcium homeostasis, the main mitochondrial function is to produce ATP through oxidative phosphorylation. Mitochondrial functionality generally depends on two important components: respiratory capacity and the degree of coupling between oxidation of energy substrates and oxidative phosphorylation. A high respiratory capacity enables the cell to efficiently oxidize energy substrates and produce large quantities of ATP, which is needed to carry out all those processes that underpin cell survival. Thus, a decreased oxidative capacity can compromise the proper functioning of the cell. From this perspective, oxygen consumption represents one of the most informative parameters for assessing the organelle’s overall functionality. For this reason, tissues that require considerable energy, such as the nervous system, heart and musculature, have a particularly active mitochondrial metabolism. Therefore, it is not surprising that neurological, metabolic, cardiac and oncological diseases are increasingly associated with mitochondria dysfunction. 

Therefore, we believe that an accurate understanding of the specific mechanisms underlying mitochondrial malfunctioning is particularly important for the potential development of any pharmacological strategy aimed at restoring the organelle functions. This issue aims to gather recent studies on mitochondrial function under physiological and/or pathological conditions.

The Special Issue “Mitochondrial Respiration in Physiology and Pathology” of the *International Journal of Molecular Sciences* comprises a total of six contributions, five of which are articles and one of which is a review [1], providing an overview of the molecular mechanisms controlling the mitochondrial dynamics by fission, fusion, and mitophagy. Kabra and Jastroch [1] also discuss the role of mitochondrial dynamic dysfunction in pancreatic β-cells and of pharmaceutical agents (like: dynasore, a dynamin inhibitor; P110, a Drp1 inhibitor; and 15-oxospiramylactone) acting on proteins that control mitochondrial dynamics, such as mitochondrial division inhibitor-1 (Mdivi-1).

Following up on their previous observations, Scrima et al. [2] focus on the important role played by the mitochondrial sAC-cAMP-PKA axis in the modulation of ΔΨm-dependent control coefficients of respiratory chain complexes. The authors show that cAMP/PKA-mediated signaling influences the aggregation state of the respiratory complexes, highlighting that reversible phosphorylation of respiratory chain complexes/supercomplexes influences their assembly state in response to the membrane potential. A conclusion supported by examination of the available structure of the CI/CIII/CIV respirasome, in which several serine residues are exposed on the matrix side of the complexes, suggests that they are possible targets of PKA.

Magrì et al. [3] focus on the Voltage-Dependent Anion-selective Channel isoform 1 (VDAC1), the most abundant protein of the outer mitochondrial membrane (OMM) and the principal pore controlling the permeation of metabolites responsible for mitochondrial functions. In the near-haploid human cell line HAP1, the authors study the effect of VDAC1 knockout on mitochondrial respiration. Their results indicate that the lack of VDAC1 leads to a drastic reduction in oxygen consumption and a reorganization of the relative contributions of electron transport chain (ETC) enzymes. In particular, they show that complex I-related respiration (N pathway) increases by drawing resources from respiratory reserves. Thus, the contribution of VDAC1 to the cell’s entire energy metabolism is evident, confirming previous data from the same group.

Chung et al. [4] analyze the effects of modulation of mitochondrial dynamics on the poor neurological prognosis of subarachnoid haemorrhage (SAH) resulting from hyperglycaemia. The authors report the therapeutic effect of Mdivi-1, a mitochondrial division inhibitor acting on Drp-1, on early and late brain injury from experimental SAH. Specifically, under the experimental conditions studied, they show that Mdivi-1 attenuates cerebral vasospasm, adverse neurological outcomes, inflammation and apoptosis of neuronal cells.

Barath et al. [5] focus on the side effects that ceftriaxone and rifaximin, first-choice antibiotics in gastrointestinal surgery, produce on mitochondrial function. The authors show that treatment with these two antibiotics seriously impairs oxidative phosphorylation capacity (OxPhos) and leak respiration in the ileal and colon mucosa. Therefore, these results represent evidence that clinical treatment with ceftriaxone or rifaximin, although undoubtedly effective on intestinal bacteria, produces severe mitochondrial dysfunction in intestinal mucosal cells.

Finally, Zhang et al. [6] report that the enzyme gamma-aminobutyrate transaminase (ABAT), specific to cardiomyocytes and present in the mitochondrial matrix, counteracts organelle dysfunction by protecting cardiomyocytes from lipid overload-induced death. Considering the known role of ABAT in the formation of key intermediates of the tricarboxylic acid cycle [7] and together with more recent data showing its involvement in mitochondrial biogenesis [8], the authors suggest that ABAT plays a role in cardiac pathology representing a valuable therapeutic target for cardiac damage associated with lipid overload.
